# Improving the Biocompatibility of Plant-Derived Scaffolds for Tissue Engineering Using Heat Treatment

**DOI:** 10.3390/jfb16100380

**Published:** 2025-10-10

**Authors:** Arvind Ramsamooj, Nicole Gorbenko, Cristian Olivares, Sashane John, Nick Merna

**Affiliations:** 1 Fred DeMatteis School of Engineering and Applied Science, Hofstra University, Hempstead, NY 11549, USA; aramsamooj1@pride.hofstra.edu (A.R.); ngorbenko1@pride.hofstra.edu (N.G.); colivares1@pride.hofstra.edu (C.O.); sjohn2@pride.hofstra.edu (S.J.); 2 Cardiothoracic Surgery, Northwell Health, New York, NY 11030, USA

**Keywords:** decellularization, plant-derived scaffolds, biocompatibility, tissue engineering

## Abstract

Small-diameter vascular grafts often fail due to thrombosis and compliance mismatch. Decellularized plant scaffolds are a biocompatible, sustainable alternative. Leatherleaf viburnum leaves provide natural architecture and mechanical integrity suitable for tissue-engineered vessels. However, the persistence of immunogenic plant biomolecules and limited degradability remain barriers to clinical use. This study tested whether mild heat treatment improves scaffold biocompatibility without compromising mechanical performance. Decellularized leatherleaf viburnum scaffolds were treated at 30–40 °C in 5% NaOH for 15–60 min and then evaluated via tensile testing, burst pressure analysis, scanning electron microscopy, histology, and in vitro assays with white blood cells and endothelial cells. Scaffold properties were compared to those of untreated controls. Heat treatment did not significantly affect scaffold thickness but decreased fiber area fraction and diameter across all anatomical layers. Scaffolds treated at 30–35 °C for ≤30 min retained >90% of tensile strength and achieved burst pressures ≥820 mmHg, exceeding physiological arterial pressures. Heat treatment reduced surface fractal dimension while increasing entropy and lacunarity, producing a smoother but more heterogeneous microarchitecture. White blood cell viability increased up to 2.5-fold and endothelial cell seeding efficiency improved with treatment duration, with 60 min producing near-confluent monolayers. Mild alkaline heat treatment therefore improved immune compatibility and endothelialization while preserving mechanical integrity, offering a simple, scalable modification to advance plant-derived scaffolds for grafting.

## 1. Introduction

Small-diameter vascular grafts (<6 mm) are in high demand for coronary and peripheral bypass surgeries, yet their clinical performance remains suboptimal [1,2]. Autologous vessels, such as the saphenous vein, are the preferred grafts, but roughly 20% of patients lack suitable vein grafts due to prior harvests or vascular disease [3,4,5]. Synthetic grafts, such as ePTFE or Dacron, are often used as alternatives, but these have low patency in small arteries and often fail because of thrombosis and inflammation driven by compliance mismatch and lack of endothelium [6,7].

Decellularized plant tissues have emerged as a novel platform for small-diameter vascular graft engineering in recent years [8,9]. Our prior studies with leatherleaf viburnum (*Viburnum rhytidophyllum*) have reported reduced thrombosis after endothelialization and bioreactor conditioning under whole-blood perfusion, with higher luminal endothelial density, suture retention at transplantation thresholds, and preserved material strength. Plant-derived scaffolds are primarily composed of cellulose, a biocompatible polysaccharide that can confer mechanical properties comparable to certain human tissues [10]. These scaffolds are also inexpensive, abundant, and free from donor scarcity and ethical concerns associated with human or animal graft materials. Modulevsky et al. showed that decellularized apple tissue supports mammalian cell ingrowth in vitro and provokes only a mild, transient immune response in vivo [10]. Similarly, Gershlak et al. recellularized spinach leaves with human endothelial cells (ECs), capitalizing on the leaf’s native vasculature to create perfusable tissue constructs [11]. These pioneering efforts highlight the promise of cross-kingdom tissue engineering, using plant-derived scaffolds as biomaterials for vascular applications.

Here, we studied decellularized leatherleaf scaffolds to test how mild heat treatment affects their structure, mechanics, and cell compatibility, building on our prior work, which involved forming tubes with endothelialized luminal surfaces and a non-thrombogenic lining. Our previous scanning electron microscopy (SEM) comparisons showed a smoother adaxial (upper) leaf face and a trichome-rich abaxial (lower) face, which motivated us to use the adaxial surface as the lumen

Despite the advantages of plant-based scaffolds, there are two primary hurdles to their use in vivo: limited degradability and potential immunogenicity. First, cellulose is not readily broken down in the human body due to the absence of cellulolytic enzymes, so a decellularized plant scaffold may persist long-term without being resorbed. Prolonged persistence of the graft can impede integration with host tissue and may provoke chronic foreign body reactions. Second, residual plant biomolecules can trigger immune responses. Residual plant proteins, such as lectins, may activate immune pathways, provoking an early inflammatory response, although some studies have suggested that this subsides over time [10]. In this study, heat treatment of the scaffold is explored to mitigate both issues. Heat treatment may reduce immunogenicity by denaturing residual proteins while preserving cellulose structure, offering a milder alternative to chemical or enzymatic methods. Boiling plant tissue is known to inactivate the majority of lectin activity, suggesting that a controlled, moderate heat treatment might reduce scaffold immunogenicity without introducing the cytotoxic residues associated with chemical or enzymatic treatments [12].

To date, however, there is little quantitative data on how such heat treatments affect the extracellular matrix (ECM) morphology, mechanical properties, and immune compatibility of decellularized plant scaffolds. Decellularization protocols, including a high-temperature step at 95 °C, have been used to strip parenchymal tissue from leaves [13]; hwoever, no studies have systematically evaluated the efficacy of milder heat treatments in tuning scaffold degradation while preserving the integrity of the plant-derived vascular structure. This knowledge gap limits the development of plant-based vascular grafts.

Recent work has shown rapid progress with decellularized plant tissues across multiple applications. Cellulose scaffolds are biocompatible, inexpensive, and naturally porous, with low thrombogenicity and tunable mechanics, which has generated interest in their use for vascular applications [14]. Examples include 3D neural stem cell culture on asparagus-derived cellulose [15], human skeletal muscle formed on biofunctionalized leaf substrates with oriented myotubes and contraction [16], and floral bract scaffolds that support skin cell adhesion and differentiation without the need for added motifs [17]. Other studies have explored applications for bone [18,19], cartilage [20], dentistry [21], and other soft and hard tissues [22]. For vascular targets, decellularized leatherleaf reinforced with gelatin yielded small-diameter grafts that supported endothelialization and exhibited suitable tensile and rupture properties [8]. Subsequent perfusion conditioning further increased luminal endothelial density and reduced thrombus formation under physiological pressures [23]. These advances motivate the present study on heat-treated plant scaffolds for improved biocompatibility and handling.

This study investigated the use of mild alkaline heat treatment to improve the structural and biological performance of plant-derived scaffolds for vascular tissue engineering. Using leatherleaf viburnum, we examined how controlled heat exposure affected ECM structure, mechanical strength, and cell compatibility. Heat treatment enhanced white blood cell (WBC) viability and endothelialization while preserving tensile strength and burst pressure. These findings demonstrate that a simple, scalable thermal modification can mitigate immunogenicity and support endothelial lining, advancing the development of plant-based scaffolds for small-diameter vascular grafts.

## 2. Materials and Methods

### 2.1. Heat Treatment and Decellularization of Plant Leaves

**Figure 1:**
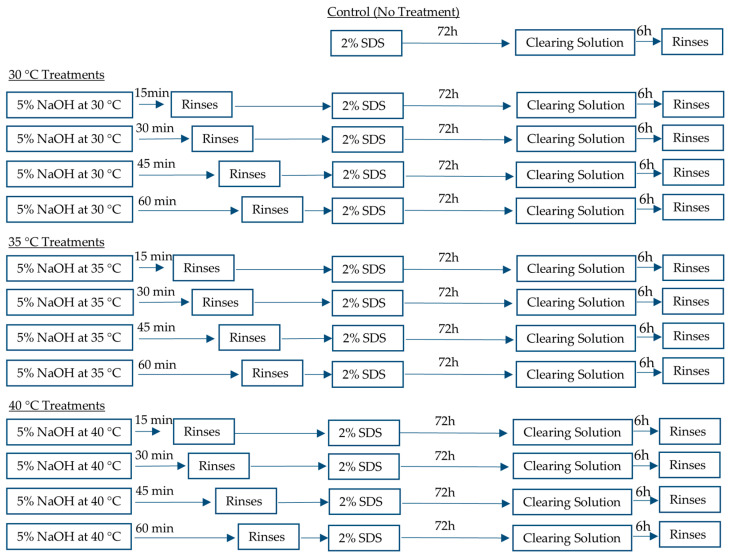
Schematic of heat treatment and decellularization protocols applied to leatherleaf viburnum. Leatherleaf treated at 30 °C, 35 °C, or 40 °C in 5% NaOH for 15, 30, 45, or 60 min, followed by decellularization in 2% SDS and clearing solution.

Fresh leatherleaf viburnum obtained from a single location was rinsed in deionized water and treated at 30 °C, 35 °C, or 40 °C in 5% NaOH for 15, 30, 45, or 60 min (Figure 1) in a fume hood (Labconco Corporation, Kansas City, MO, USA). Leaves were then decellularized with 2% sodium dodecyl sulfate (SDS) for 72 h in an orbital shaker at 37 °C, followed by clearing solution (0.1% Triton X-100 and 10% bleach) for 6 h. Samples were sterilized in 70% ethanol for 10 min and stored in phosphate-buffered saline (PBS) for up to two weeks at 4 °C. Control samples underwent the same decellularization and sterilization steps but did not receive heat treatment. Additional exploratory samples were treated at higher temperatures (50 °C, 70 °C, and 90 °C for 60 min) to qualitatively observe the effects of more aggressive conditions; these were not included in the quantitative analyses.

### 2.2. DNA Quantification

Leatherleaf samples (30–50 mg per condition; *n* = 3) were weighed, homogenized with FastPrep-24 Lysing Matrix A (MP Biomedical, Irvine, CA, USA), and digested in lysis buffer containing proteinase K (Omega Bio-Tek, Norcross, GA, USA) for 30 min at 56 °C. DNA was extracted using magnetic beads (Omega Bio-Tek) and quantified photometrically at 260 nm, as previously described [8]. Percent DNA removal was calculated relative to the DNA content of non-decellularized leatherleaf samples.

### 2.3. Tensile Testing

Dog bone-shaped samples were cut from decellularized leatherleaf viburnum for each heat treatment condition (*n* = 3). Gauge thickness, width, and length were recorded for each sample. Uniaxial tension was applied at 0.08 mm/s until failure, as previously described [24]. Maximum tensile load and elastic modulus were calculated from the load and extension data.

### 2.4. Burst Pressure Testing

Decellularized leatherleaf for each heat treatment group was trimmed to 25 mm × 21 mm and wrapped around a 1.5 mm diameter stainless steel rod, with the adaxial side facing inward. A mixture of 100 μL of 50% gelatin heated to 55 °C and 20 μL of 25% glutaraldehyde (Alfa Aesar, Ward Hill, MA, USA) was applied evenly along the leaf’s width. The remaining portion was wrapped around the rod and held for 1 min to allow cross-linking. Constructs were incubated overnight at 37 °C. These grafts were then secured to barb adapters using rubber bands, with one end attached to a pressure sensor (Automation Products Group, Logan, UT, USA). Water was injected into the opposite end at constant pressure using a syringe, and a slow-motion camera recorded peak pressure at failure, as previously described [8]. Graft compliance was measured according to ANSI/ISO 7198:2016 from pressure and diameter measurements, as previously described [25,26].

### 2.5. Scanning Electron Microscopy

Decellularized leatherleaf samples for each heat treatment group were fixed and dehydrated with a graded ethanol series (70–100%) for 1 h. Samples were dried using a Samdri-795 critical point dryer (Tousimis, Rockville, MD, USA), mounted on aluminum stubs, and coated with gold using an EMS-550 sputter coater (Electron Microscopy Sciences, Hatfield, PA, USA). A Quanta FEI-250 scanning electron microscope (Thermo Fisher Scientific, Waltham, MA, USA) was used to image abaxial and adaxial surfaces at 200× and 800× magnification.

Images at 200× magnification were converted to binary and 8-bit formats using ImageJ version 1.54g (Wayne Rasband and contributors, National Institutes of Health, Bethesda, MD, USA). Fractal dimension and lacunarity were calculated using the box-counting method with the FracLac plugin version 2015Sep090313a9330 to assess surface roughness and heterogeneity, respectively. Contrast and entropy were measured with the GLCM Texture Analyzer plugin version 0.4 to assess the complexity and randomness of the surface, as previously described [27]. Three images per sample were analyzed (*N* = 9 per test).

### 2.6. Histological Staining

Decellularized leatherleaf for each heat treatment group was fixed in 10% formaldehyde (Sigma-Aldrich, St. Louis, MO, USA) for 30 min, dehydrated in a graded ethanol series (70–100%) for 2 h, rinsed with xylene for 1 h, and embedded in paraffin wax (Sigma-Aldrich, St. Louis, MO, USA) for 24 h at 72 °C and under a vacuum at 0 mmHg. Samples were vertically embedded in metal molds and stored at 4 °C. Paraffin blocks were sectioned at 8 µm, mounted on charged microscope slides, deparaffinized with xylene, and rehydrated through a descending ethanol series (100–50%), as previously described [23]. Sections were stained for 10 min with a 0.1% Safranin-O solution (Aldon, Avon, NY, USA), rinsed, and mounted using Histomount (National Diagnostics, Atlanta, GA, USA). Brightfield images were captured at 10× magnification (Olympus FV3000 microscope, Tokyo, Japan). Fiber thickness for the upper epidermis, palisade mesophyll, spongy mesophyll, and lower epidermis, as well as the fiber area fraction, were measured using ImageJ for 3 biological replicates per condition, with 4 measurements per region.

### 2.7. White Blood Cell Assay

Fresh rat blood was collected in citrate tubes and kept at room temperature for 1 h. A total of 4 mL of blood was diluted with 4 mL of PBS and layered on top of Lymphoprep Density Gradient Medium (STEMCELL Technologies, Vancouver, BC, Canada). Samples were centrifuged at 400× *g* for 30 min at 18 °C. The mononuclear cell layer was collected, washed in PBS, centrifuged, and resuspended in M199 medium. To assess the in vitro foreign body response, 200,000 WBCs were seeded onto 1 cm × 1 cm decellularized leatherleaf viburnum scaffolds under each heat treatment condition (*n* = 3). After 24 h, cell density was measured using trypan blue exclusion and compared to polystyrene controls as previously described [28].

### 2.8. Recellularization of Plant-Derived Scaffolds

ECs were seeded onto the adaxial surface of scaffold sheets, which corresponds to the luminal surface in our previously described rolled tubular constructs. Primary rat aortic ECs (Cell Applications, San Diego, CA, USA) were expanded at 5% CO_2_ and used up to passage 7. Cells were cultured in Rat EC Growth Medium (Cell Applications, San Diego, CA, USA) supplemented with 2% fetal calf serum, hydrocortisone, endothelial growth supplement, basic fibroblast growth factor, epidermal growth factor, and heparin. Decellularized leaves under each heat treatment were coated with 20 μg/ml fibronectin (Sigma-Aldrich, St. Louis, MO, USA) for 1 h. ECs were seeded at a density of 625,000 cells/cm^2^ onto the adaxial surface of the leaf and incubated for 5 h before feeding. After 24 h, scaffolds were rinsed in PBS, fixed in 4% formaldehyde, and stained with Hoechst nuclear stain for 30 s. Nuclei were manually counted in three confluent regions per sheet (*n* = 3) by blinded counters using fluorescence microscopy (Olympus FV3000, Tokyo, Japan) at 20× magnification.

### 2.9. Statistical Analysis

Anderson–Darling tests were used to assess normality. For comparisons involving three or more groups, one-way analysis of variance (ANOVA) was used. When significant differences were found, Tukey’s post hoc test was applied for pairwise comparisons. Statistical significance was defined as *p* < 0.05. All analyses were performed in Microsoft Excel version 2508 (Redmond, WA, USA). Data are presented as mean ± standard deviation. Sample sizes (*N* = 3) refer to biological replicates unless otherwise specified.

## 3. Results

### 3.1. Assessment of Decellularization

**Figure 2:**
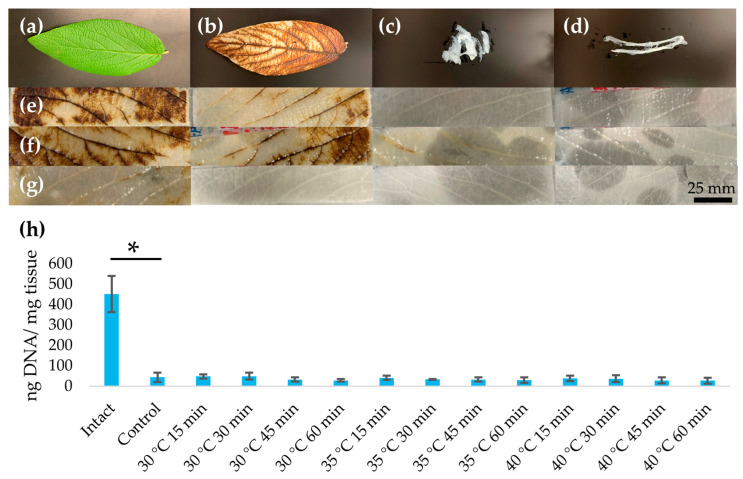
Representative images of (**a**) non-decellularized leatherleaf, and exploratory high-temperature treatments to illustrate structural damage under aggressive conditions (**b**) 50 °C, (**c**) 70 °C, or (**d**) 90 °C in 5% NaOH for 60 min, followed by decellularization in 2% SDS and clearing solution. Leatherleaf treated at (**e**) 30 °C, (**f**) 35 °C, or (**g**) 40 °C in 5% NaOH for 15, 30, 45, or 60 min, followed by decellularization in 2% SDS and clearing solution, and (**h**) resulting DNA content. * *p* < 0.05 and error bars represent standard deviation (*n* = 3).

Decellularized leatherleaf samples appeared increasingly opaque and whitish with increasing heat treatment times (Figure 2). To provide context for the effects of more extreme protocols, we show representative images of our exploratory treatments at 50–90 °C, which caused substantial whitening and tissue damage (Figure S1) that was not seen under the milder conditions used in this study. Despite these changes in appearance under milder conditions, quantitative measurements of scaffold thickness revealed no significant differences across heat treatment times or temperatures (*p* > 0.1). All decellularization conditions resulted in ≥90% DNA removal with <50 ng DNA/ mg tissue (Table 1).

### 3.2. Mechanical Properties 

**Figure 3:**
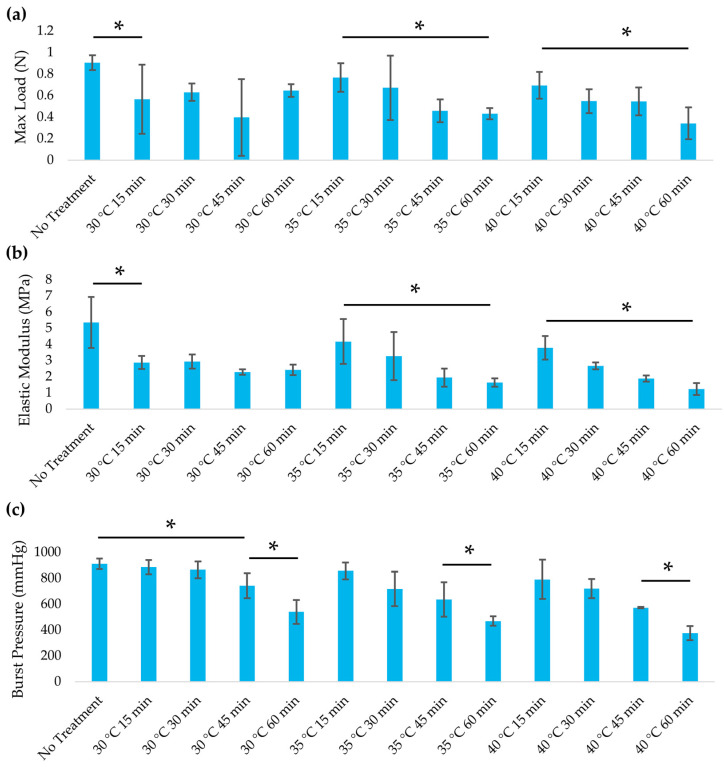
(**a**) Maximum load and (**b**) elastic modulus for leatherleaf treated at 30 °C, 35 °C, or 40 °C in 5% NaOH for 15, 30, 45, or 60 min, followed by decellularization in 2% SDS and clearing solution. (**c**) Burst pressure of 3D grafts created using scaffolds treated at 30 °C, 35 °C, or 40 °C in 5% NaOH for 15, 30, 45, or 60 min. * *p* < 0.05 and error bars represent standard deviation (*n* = 3).

Mild alkaline heat treatments had minimal impact on the mechanical properties of the decellularized leatherleaf scaffold, whereas more aggressive conditions led to significant weakening (Figure 3). In tensile tests, scaffolds treated at 30 °C or 35 °C for 15 to 30 min retained elasticity and tensile strength that were statistically comparable to untreated decellularized controls (*p* > 0.05). By contrast, prolonged exposure or higher temperature caused a measurable drop in stiffness and load-bearing capacity (*p* < 0.05). Treatment at 40 °C for 60 min reduced the elastic modulus by 43% relative to the 30 °C, 15 min condition. The maximum tensile load at failure showed a similar trend, dropping significantly under the most extreme heat protocol. Moderate heat treatments preserved ≥90% of the initial mechanical strength, indicating that temperatures of 30–35 °C for short durations are optimal for maintaining scaffold integrity. Notably, the modest reductions in stiffness observed after longer treatments brought the scaffold’s elastic modulus closer to that of a native coronary artery (1.5 MPa), potentially improving compliance matching [29]. All groups exhibited a similar order of magnitude of stiffness, which was far below the rigidity of synthetic ePTFE grafts and more closely matches the compliance of natural blood vessels (Table 2). Leaf thickness measurements confirmed that no gross dimensional changes occurred with these mild heat treatments (*p* > 0.1), suggesting the structural alterations were primarily biochemical (partial delignification) rather than macroscopic shrinkage. These results demonstrate that an appropriate window of mild heat treatment can tune the scaffold’s mechanical properties, achieving a balance between preserving strength and enhancing compliance.

Burst pressure testing revealed that vascular graft mechanics declined with more aggressive heat exposure. Under mild conditions (30–35 °C for ≤30 min), the grafts retained burst pressures comparable to those of untreated controls (*p* > 0.05). However, longer or higher-temperature treatments led to significant reductions. At 30 °C, a notable drop in burst pressure was first observed after 45 min of heating (*p* < 0.05 vs. no-heat control), whereas at 40 °C, a significant decline occurred after only 15 min (*p* < 0.05). By 60 min of treatment, all temperature groups showed a pronounced decrease in burst pressure relative to untreated scaffolds (*p* < 0.05). These results indicate that mild heat treatments preserved the graft’s ability to withstand intraluminal pressure (no significant drop in burst pressure vs controls), whereas more extreme protocols substantially compromised it.

### 3.3. Structural Analysis 

**Figure 4:**
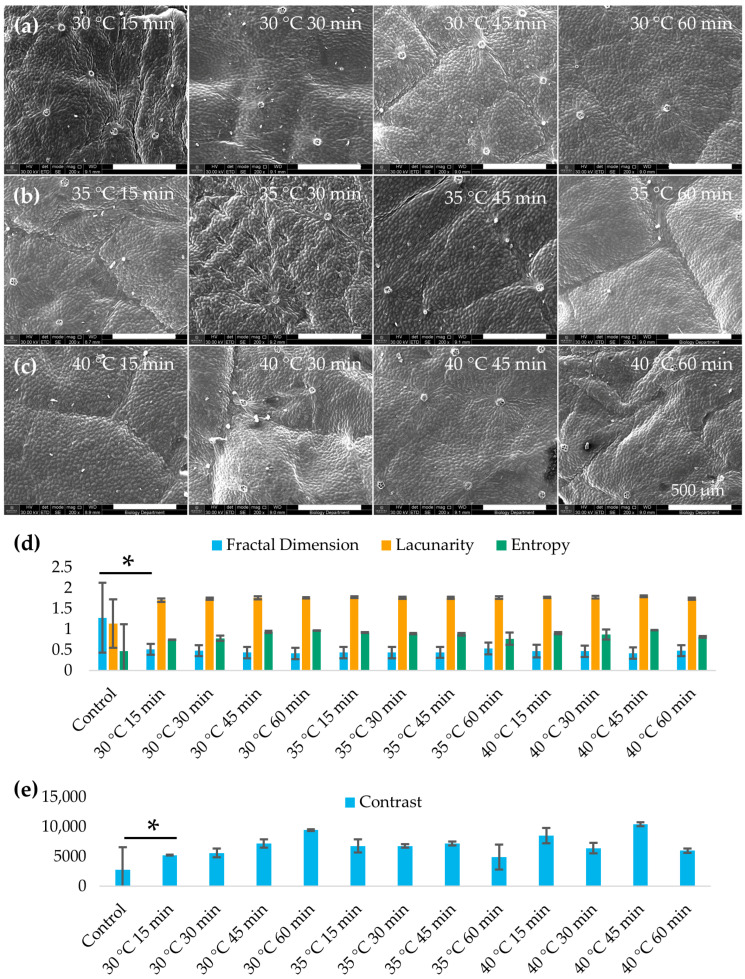
Leatherleaf treated at (**a**) 30 °C, (**b**) 35 °C, or (**c**) 40 °C in 5% NaOH for 15, 30, 45, or 60 min, followed by decellularization in 2% SDS and clearing solution, and imaged by SEM at 200× magnification (*n* = 3). (**d**) Fractal dimension, lacunarity, entropy, and (**e**) contrast were measured for each condition for 3 images per sample (*n* = 9). * *p* < 0.05 and error bars represent standard deviation.

SEM image analysis revealed that mild alkaline heat treatment noticeably altered the scaffold’s microarchitectural texture (Figure 4). Heat-treated leatherleaf viburnum scaffolds exhibited a significantly lower fractal dimension than untreated controls (*p* < 0.05), indicating a reduction in micro-scale surface complexity. Lacunarity increased with heat treatment (*p* < 0.05), consistent with a more heterogeneous distribution of surface features. Image texture metrics (grayscale contrast and entropy) also increased in the treated group (*p* < 0.05), reflecting greater pixel intensity variation and randomness. Together, these changes suggest that while heat treatment smooths the overall surface (decreasing fine fractal roughness), it simultaneously introduces a more irregular, patchy topology compared to the untreated scaffolds.

**Figure 5:**
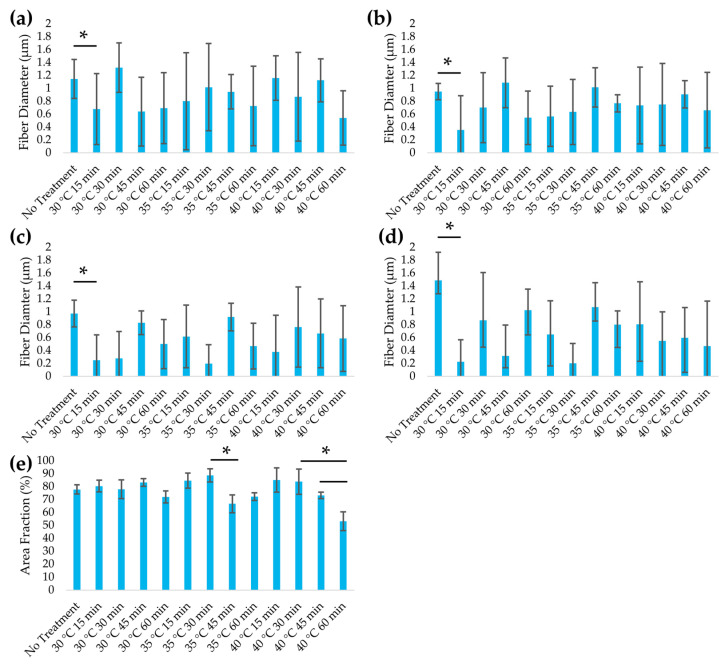
Fiber diameter in the (**a**) upper epidermis, (**b**) palisade mesophyll, (**c**) spongy mesophyll, and (**d**) lower epidermis, and (**e**) fiber area fraction, of leatherleaf treated at 30 °C, 35 °C, or 40 °C in 5% NaOH for 15, 30, 45, or 60 min, followed by decellularization in 2% SDS and clearing solution. * *p* < 0.05 and error bars represent standard deviation (3 images per sample, *n* = 9).

Heat treatment significantly reduced fiber diameter across all regions of the leaf scaffold (*p* < 0.05), indicating partial structural degradation. Leatherleaf ECM microstructures were quantitatively assessed through histology (Figure S2) and the measurement of average fiber thickness (Figure 5) across four regions: the upper epidermis, palisade mesophyll, spongy mesophyll, and lower epidermis. In the lower epidermis, the average fiber diameter decreased from 1.5 to 0.6 µm (58%) across the various heat treatment conditions. Reductions were also observed in the upper epidermis (1.1 to 0.9 µm), palisade mesophyll (1.0 to 0.7 µm), and spongy mesophyll (1.0 to 0.5 µm). These findings indicate that heat treatment alters ECM structure in all regions, with the lower epidermis showing the greatest change. Fiber area fraction declined with increasing temperature and time. Within each temperature series, the 60 min group exhibited a lower fiber area fraction than the 15 min group (*p* < 0.05). At 60 min, the 40 °C group exhibited a lower fiber area fraction than the 30–35 °C group (*p* < 0.05), reflecting increased porosity at the most severe heat treatments, which is consistent with the observed reduction in fiber diameter.

### 3.4. Assessment of Immunogenicity 

**Figure 6:**
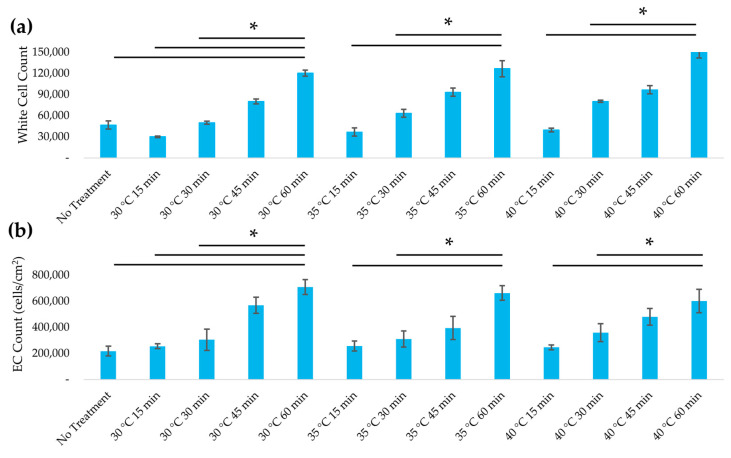
(**a**) White blood cell viability and (**b**) EC count for leatherleaf treated at 30 °C, 35 °C, or 40 °C in 5% NaOH for 15, 30, 45, or 60 min, followed by decellularization in 2% SDS and clearing solution. * *p* < 0.05 and error bars represent standard deviation. Cells were counted using 3 images per sample (*n* = 9).

Heat-treated plant scaffolds supported WBCs with markedly higher viability in vitro, indicating a reduction in cytotoxic or immunogenic components after treatment. Figure 6 shows that WBC viability increased progressively with longer heat exposure times. Scaffolds treated for 60 min exhibited the highest viability, with a 2.5-fold increase compared to untreated scaffolds (no heat) (*p* < 0.05). In contrast, varying the treatment temperature among 30 °C, 35 °C, and 40 °C at a given time point had relatively minor effects on viability. Increasing the duration from 15 min to 60 min at 35 °C increased viable WBC counts by three-fold, whereas increasing the temperature from 30 °C to 40 °C (at 60 min) only yielded a 27% increase in viability (*p* < 0.05). The highest viability was achieved on scaffolds treated for 60 min (regardless of temperature), a significant improvement over the viability on untreated decellularized controls (*p* < 0.05). This data indicates that extended mild heating in 5% NaOH significantly attenuates the scaffold’s acute cytotoxicity. We suspect that this improvement is due to the denaturation or removal of residual plant biomolecules that can trigger immune cell stress, such as plant-derived lectins and other proteins known to cause immune cell aggregation and toxicity if not inactivated. These findings suggest that the 5% NaOH heat protocol effectively improves the WBC compatibility of the plant scaffold.

### 3.5. Recellularization of Decellularized Leatherleaf with ECs 

**Figure 7:**
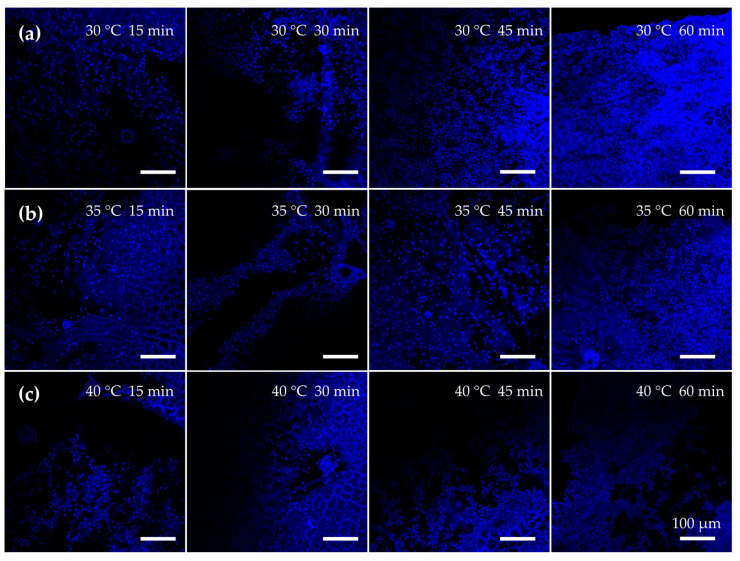
Leatherleaf treated at (**a**) 30 °C, (**b**) 35 °C, or (**c**) 40 °C in 5% NaOH for 15, 30, 45, or 60 min, followed by decellularization in 2% SDS and clearing solution, was seeded with endothelial cells, stained with Hoechst after 24 h, and imaged at 20× magnification. Three images were collected per sample (*n* = 9).

Heat treatment significantly enhanced EC seeding efficiency and the formation of an endothelial monolayer on the scaffold surface. These cell-seeding results model the endothelialization of the adaxial luminal surface used in our rolled tubes previously [8,23]. After 24 h in static culture, EC attachment was observed on all scaffolds; however, those subjected to longer heat treatments exhibited substantially higher cell densities. Scaffolds treated at 30 °C for 60 min supported the greatest EC coverage, with an average surface cell density of approximately 708,000 cells/cm^2^, which was 178% higher than that on scaffolds treated for 15 min (*p* < 0.05). The untreated and minimally treated scaffolds showed patchy EC coverage with some bare regions, whereas the 45 min and 60 min heat-treated groups achieved near-uniform coverage (Figure 7). By contrast, scaffolds with no heat or only 15 min treatment had noticeably sparser nuclei and incomplete coverage. Increasing the treatment temperature from 30 °C to 40 °C did not significantly affect EC seeding; however, all three temperature groups showed improved EC attachment with longer durations. These results highlight that treatment duration was the dominant factor for promoting endothelialization and that heat treatment significantly improved the scaffold’s ability to be recellularized with ECs, achieving a near-confluent monolayer in vitro.

## 4. Discussion

This study demonstrates that applying a mild heat treatment to decellularized leatherleaf viburnum scaffolds (30–40 °C, 5% NaOH, ≤60 min) improves their biocompatibility without compromising structural performance. We tested leaves as sheets to assess scaffold changes, noting that the intended application is a rolled tube with the adaxial surface as the endothelialized lumen. Moderate heat preserved >90% of the scaffold’s mechanical strength while reducing stiffness to better match native coronary artery compliance. Heat treatment significantly increased WBC viability by up to 2.5-fold compared to untreated controls. All treatments met the accepted decellularization benchmark of ≤50 ng DNA/mg tissue, confirming the effective removal of cellular remnants and limiting nucleic acid-driven inflammation [34]. The enhanced DNA reduction after 45–60 min may help explain the improved WBC viability, offering a biocompatibility gain without altering the scaffold thickness. EC seeding efficiency improved with duration, with 60 min treatments supporting near-confluent monolayers. These enhancements suggest that heat treatment reduces acute immune responses and promotes endothelialization, two key challenges in the development of functional small-diameter vascular grafts. Thus, heat treatment offers a simple and scalable method for tailoring plant-derived scaffolds for in vivo vascular grafting.

Our results fit a growing body of literature that positions plant-derived scaffolds as credible candidates for vascular grafts. Prior leatherleaf–gelatin grafts supported endothelialization and maintained tensile and rupture strength [8]. Meanwhile, perfusion bioreactor conditioning further increased endothelial coverage and reduced thrombus formation under pulsatile flow and pressure cycles ranging from 50 to 120 mmHg [23]. Beyond vessels, recent studies in neural, muscle, and skin systems have demonstrated that plant microarchitecture supports mammalian cell growth and maturation, including the formation of oriented myotubes with contractile function on leaf substrates [15,16,17]. Method papers now call for clearer reporting and standardization of plant-based scaffold processing, which we follow here when describing heat treatment, decellularization, and DNA removal [14,24]. Imaging improvements, such as autofluorescence quenching, can also aid analysis without altering mechanics and can be cited where relevant to your microscopy [35].

The preservation of tensile strength and elasticity under mild heat treatment is an encouraging outcome that addresses a key design criterion for vascular grafts. Our results showed that treating leatherleaf scaffolds at 30–35 °C for short durations yields mechanical properties similar to those of untreated scaffolds, whereas more aggressive treatments caused a moderate decline in stiffness. This finding is consistent with observations in decellularized scaffolds, where overly harsh processing can damage matrix integrity [13,36]. In the context of vascular grafts, maintaining appropriate compliance is crucial: a graft that is stiffer than the native artery can create stress concentrations at the anastomoses, leading to intimal hyperplasia and graft failure [37,38]. By fine-tuning the heat exposure, we produced scaffolds with an elastic modulus approaching that of native coronary arteries, which may mitigate well-known compliance mismatch issues. Notably, the treated plant grafts remain far more compliant than synthetic ePTFE grafts, which have orders-of-magnitude higher modulus and contribute to poor long-term patency. Therefore, a slight reduction in stiffness due to partial removal of hemicellulose or lignin during treatment could be beneficial as long as the scaffold retains sufficient strength for surgical handling. In our case, even the most degraded samples withstood tensile loads comparable to native vessels, well above suture retention requirements [39,40]. Our findings demonstrate that a mild heat pre-treatment can adjust scaffold stiffness within a physiologically relevant range without severely compromising tensile integrity. This balance is promising, as it suggests the graft can better match the mechanical behavior of host vessels while still enduring implantation and blood flow.

Heat treatment of decellularized leatherleaf viburnum scaffolds led to a notable decrease in fiber diameter across all four leaf regions (upper epidermis, palisade mesophyll, spongy mesophyll, and lower epidermis). This uniform fiber thinning supports the notion that thermal processing in alkaline solution partially degrades the cellulose-rich ECM structure. Similar observations have been reported in plant-based decellularization studies where more aggressive processing causes fiber thinning. In our study, heat treatment reduced fiber diameter in the lower epidermis by 58%, closely matching the 60% reduction reported with prolonged chemical bleaching [24], suggesting that even mild thermal exposure in alkaline conditions can produce structural alterations comparable to more aggressive decellularization protocols. Harsh alkaline or oxidative treatments (NaOH or hypochlorite bleach) are known to damage cellulose microfibrils, yielding thinner fibers and increased porosity at the cost of mechanical integrity. Notably, preserving the native ECM fiber architecture is critical for graft performance. Excessive fiber degradation can compromise mechanical stability and remove cell-adhesive cues, whereas maintaining thicker fibers has been linked to improved scaffold strength and EC repopulation. The fiber area fraction also decreased with temperature and duration, indicating greater porosity under the most severe conditions. Combined with fiber thinning, this suggests a more open and permeable scaffold that may enhance perfusion, transport, and cell infiltration, but at the risk of reduced mechanical strength. Optimal processing must therefore strike a balance between porosity and ECM integrity.

The burst pressure results mirror the tensile trends, highlighting the trade-off between scaffold strength and biocompatibility. Mild heat treatments preserved high burst pressures in our grafts, whereas harsh heat treatments significantly reduced their pressure tolerance. Grafts treated at 30–35 °C retained burst pressures similar to that of native saphenous vein (1260 ± 230 mmHg) [41], and exceeded the physiological upper limit of arterial pressure (~250 mmHg). This margin is critical for surgical handling, as a graft must endure forces from suturing, clamping, and transient pressure spikes without rupture. Although engineered grafts may not need to match arterial burst strength, studies have suggested that thresholds above 600–700 mmHg are sufficient for safe implantation [42,43]. Our mildly treated grafts surpassed this benchmark (880 mmHg). However, under the most aggressive treatment (40 °C for 60 min), burst pressure dropped significantly, indicating that excessive matrix degradation compromises structural integrity. An appropriate mild heat regimen maintains burst pressure within a clinically safe range while improving compliance and biocompatibility. These grafts are likely to withstand implantation and physiological flow while better mimicking native vessel mechanics, thereby reducing risks associated with compliance mismatch or mechanical failure.

Heat treatment that modestly increases porosity and reduces fiber diameter can accelerate endothelialization by opening inter-fiber pathways and increasing surface area; however, excessive thinning weakens the plant cellulose matrix and impairs handling. In decellularized leatherleaf scaffolds, longer clearing reduced fiber diameter and lowered endothelial seeding, whereas gentler protocols preserved microarchitecture, tensile properties, and seeding efficiency [24]. Evidence from polymer grafts supports this balance: hybrid PCL/PDS grafts that gradually increased pore openness improved EC coverage while maintaining patency in vivo, whereas very open structures risk leakage or strength loss if they are not reinforced [44]. Notably, reinforcement can safeguard handling without closing pores. For example, bacterial–cellulose grafts with an external mesh achieved full endothelial lining and 100% patency at 4 weeks in a pig coronary model with no aneurysm [45]. Finally, surgical applicability depends on maintaining mechanics within a physiological range, as compliance mismatch is a known driver of intimal hyperplasia and failure in small-diameter synthetic grafts [46].

The smoother yet more heterogeneous surfaces observed in SEM images of heat-treated scaffolds have important implications for scaffold performance. Surface morphology is known to modulate cell attachment and tissue integration. The elevated entropy and contrast of heat-treated scaffold images indicate a more complex texture that could affect endothelialization. Moderate roughness and pore sizes are often beneficial for EC attachment, providing footholds and shelter from shear stress. In synthetic grafts such as ePTFE, intermediate internodal pore sizes (~60 μm) led to greater microvessel ingrowth and endothelial coverage, whereas larger pores (~100 μm) triggered higher macrophage infiltration and impeded endothelialization [47]. Our heat-treated scaffolds’ increase in surface heterogeneity might create niches that promote initial EC attachment or migration, but an overly irregular landscape could also yield uneven cell distribution. Notably, decellularized plant scaffolds inherently present unique topographies, such as aligned fibrous ribs of celery or leaf vein networks, that can direct cell orientation and growth [48]. Altering these native patterns through post-decellularization treatment may influence how ECs organize on the lumen. Encouragingly, quantitative texture analysis tools like fractal dimension and lacunarity have been successfully employed to predict cell behavior on biomaterials [49]. For instance, fractal analysis of dental implant surfaces showed that higher surface complexity correlated with greater initial fibroblast attachment. Our finding of a reduced fractal dimension after heat treatment suggests a smoother micro-surface that might slightly lower immediate cell adhesion. In line with this finding, Massai et al. observed that increasing a scaffold’s heterogeneity via the addition of bioactive glass altered the pore architecture and was hypothesized to impact local cell–scaffold interactions [50]. Immunogenicity and thrombogenicity are also closely tied to scaffold microstructure. Studies of decellularized plant grafts implanted in animal models have reported minimal, transient inflammation [10]. The smoother surfaces observed after heat treatment may lower the adsorption of plasma proteins and platelets, an effect that may enhance in vivo hemocompatibility. Our heat-induced surface modifications can be viewed as a refinement of standard decellularization protocols, potentially decreasing immunogenic debris without compromising the cellulose scaffold’s favorable mechanical properties or its capacity for endothelialization.

The significant increase in WBC viability on heat-treated scaffolds suggests improved cytocompatibility through the inactivation of immunogenic factors. Untreated decellularized plant tissue, although primarily composed of cellulose, can still contain residual proteins and polysaccharides that provoke immune reactions. For example, plant lectins are a concern. These carbohydrate-binding proteins can trigger the lectin pathway of complement activation and cause leukocyte agglutination if not removed. Our approach of mild alkaline heat treatment appears to neutralize such components, as evidenced by the resulting increase in WBC viability compared to untreated controls. The underlying mechanism is likely protein denaturation. Shi et al. reported that boiling plant tissues for 1 h denatures most lectins (≥94% reduction in hemagglutinating activity) [12]. While our protocol (30–40 °C in NaOH) is less intense than boiling, it may still denature a substantial fraction of lectins and other proteins or facilitate their leaching out during subsequent rinses. The net effect is that immune cells experience a much more compatible environment on the treated cellulose scaffold. This result is encouraging for in vivo use, where minimizing the inflammatory response is critical to graft integration and long-term function. Prior studies have shown that decellularized cellulose induces only a transient foreign-body reaction. For example, Modulevsky et al. observed an initial macrophage infiltrate at 1 week after subcutaneous implantation of decellularized apple tissue, which then subsided completely by 8 weeks with no chronic inflammation [10]. By further minimizing immunogenic triggers, our heat-treated scaffolds could shorten the duration or intensity of that acute response, promoting faster integration. It is worth noting that an element of host immune interaction is not entirely undesirable; some infiltration by macrophages can aid in remodeling the graft, but it should be tempered and transient.

Achieving a confluent EC lining on the luminal surface of a graft is a critical milestone for vascular tissue engineering, as it provides a non-thrombogenic interface. In this study, we found that longer heat treatments significantly improved EC attachment and spreading on the decellularized plant scaffolds. The best-performing condition (60 min in NaOH) yielded a nearly confluent monolayer after 24 h, whereas scaffolds with minimal or no heat treatment had only partial coverage. These data suggest that the surface chemistry or topography of the cellulose scaffold is modified in a favorable manner by the heat treatment, perhaps by exposing more cellulose fibrils and increasing hydrophilicity after the removal of waxes, lignin, or other occluding substances. Prior research has indicated that decellularized plant matrices can inherently support endothelialization [9,11]. For example, in related work using decellularized leatherleaf scaffolds, ECs attached effectively even without the need for extrinsic ECM coatings, and the naturally smoother adaxial leaf surface was found to facilitate a more complete endothelial layer [8]. Our findings build on this knowledge, demonstrating that a brief alkaline heat pre-treatment further enhances this intrinsic EC compatibility. In comparison to synthetic graft materials, which often require surface modifications or growth factor coatings to improve EC seeding, the treated plant-derived scaffold achieved a high cell density under simple static culture conditions. This result is promising because a densely endothelialized lumen helps resist platelet adhesion and thrombosis once exposed to blood. In our prior work on endothelialized leatherleaf tubes, perfusion of whole rat blood showed markedly reduced thrombus formation after endothelialization and after bioreactor conditioning [23].

## 5. Conclusions

This study shows that mild alkaline heat treatment enhances the biocompatibility of decellularized leatherleaf viburnum scaffolds by reducing immunogenic components and improving EC attachment, while preserving key mechanical properties. Our findings support the use of controlled heat exposure as a simple, scalable modification to optimize plant-based scaffolds for vascular applications. However, the work is limited by single-donor cell testing and the absence of certain standard biocompatibility tests. Future studies will evaluate the graft with autologous ECs from ≥10 human donors to strengthen the statistical power and generalizability of endothelialization outcomes [51]. Additionally, we will conduct cytotoxicity testing in accordance with ISO 10993-5 and hemocompatibility testing as per ISO 10993-4 to confirm blood compatibility [52,53].

## 6. Patents

Provisional patent application filed: Plant-Based Vascular Grafts with Surface Modification and Dual Biophysical Stimulation and Methods of Using Same, U.S. Provisional Application No. 63/869,868, filed 25 August 2025.

## Figures and Tables

**Table 1:** DNA content of leatherleaf scaffolds under each treatment condition (*n* = 3).

Condition.	DNA (ng/mg Tissue, Mean ± SD)
Intact	452.0 ± 88.1
Control	44.5 ± 23.5
30 °C 15 min	48.6 ± 10.4
30 °C 30 min	49.3 ± 16.6
30 °C 45 min	33.1 ± 10.3
30 °C 60 min	28.8 ± 6.0
35 °C 15 min	41.8 ± 10.6
35 °C 30 min	33.8 ± 1.4
35 °C 45 min	33.2 ± 10.3
35 °C 60 min	29.8 ± 13.5
40 °C 15 min	38.3 ± 13.6
40 °C 30 min	37.3 ± 16.3
40 °C 45 min	28.6 ±14.5
40 °C 60 min	28.2 ± 14.6

**Table 2:** Mechanical properties for small-diameter grafts and native vessels.

Property	Treated Plant Scaffold	Saphenous Vein	Coronary Artery	Synthetic Grafts
Burst pressure (mmHg)	≥820	1599 [30]	2031 [31]	≥3500 [32]
Elastic modulus (MPa)	2.3–5.3	1.5–4 [33]	0.5–3 [33]	17.4 [31]
Compliance (% per 100 mmHg)	2.72	1.77 [25]	4.06 [25]	1.63 [32]

## Data Availability

The original contributions presented in the study are included in the Supplementary Materials; further inquiries can be directed to the corresponding author.

## Supplementary Materials

The following supporting information can be downloaded at https://www.mdpi.com/article/10.3390/jfb16100380/s1, Figure S1: (**a**) Maximum load and (**b**) elastic modulus for leatherleaf treated at 50 °C, 70 °C, or 90 °C in 5% NaOH for 60 min, followed by decellularization in 2% SDS and clearing solution; Figure S2: Safranin staining of leatherleaf treated at (**a**) 30 °C, (**b**) 35 °C, or (**c**) 40 °C in 5% NaOH for 15, 30, 45, or 60 min, followed by decellularization in 2% SDS and clearing solution (*n* = 3).

## Abbreviations

The following abbreviations are used in this manuscript:

NaOH	Sodium Hydroxide
ePTFE	Expanded polytetrafluoroethylene
EC	Endothelial cell
SEM	Scanning electron microscopy
ECM	Extracellular matrix
WBC	White blood cell
PBS	Phosphate-buffered saline
ANOVA	Analysis of variance
SDS	Sodium dodecyl sulfate
