# Supplementary material for: Improving the Biocompatibility of Plant-Derived Scaffolds for Tissue Engineering Using Heat Treatment
Source: J Funct Biomater. 2025 Oct 10;16(10):380. doi: 10.3390/jfb16100380 (PMC12565551; doi:10.3390/jfb16100380)
Supplement: Supplementary file 1 [file jfb-16-00380-s001.zip › jfb-3897388-supplementary.pdf]

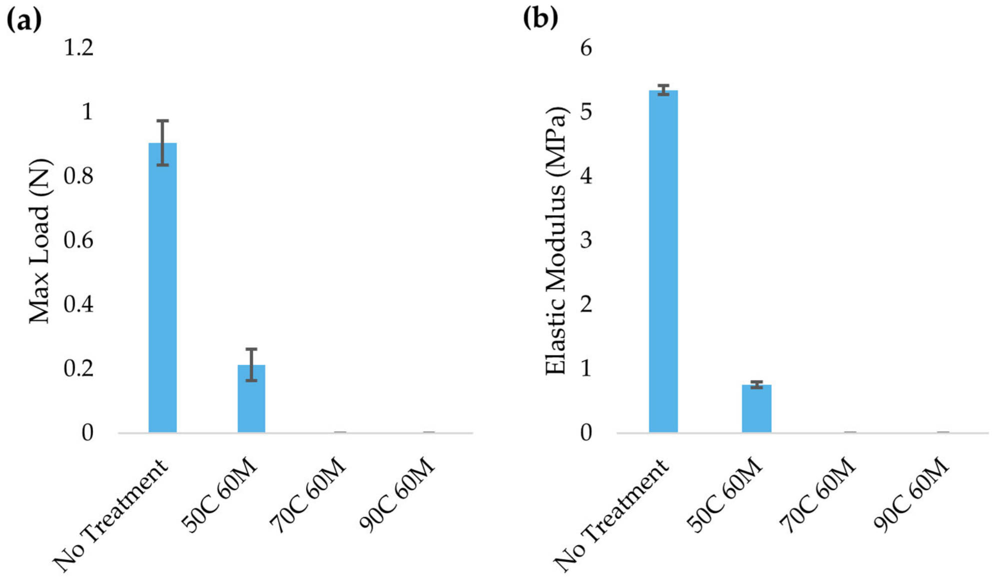


**Figure S1.** (**a**) Maximum load and (**b**) elastic modulus for leatherleaf treated at 50 °C, 70 °C, or 90 °C in 5% NaOH for 60 min, followed by decellularization in 2% SDS and clearing solution.

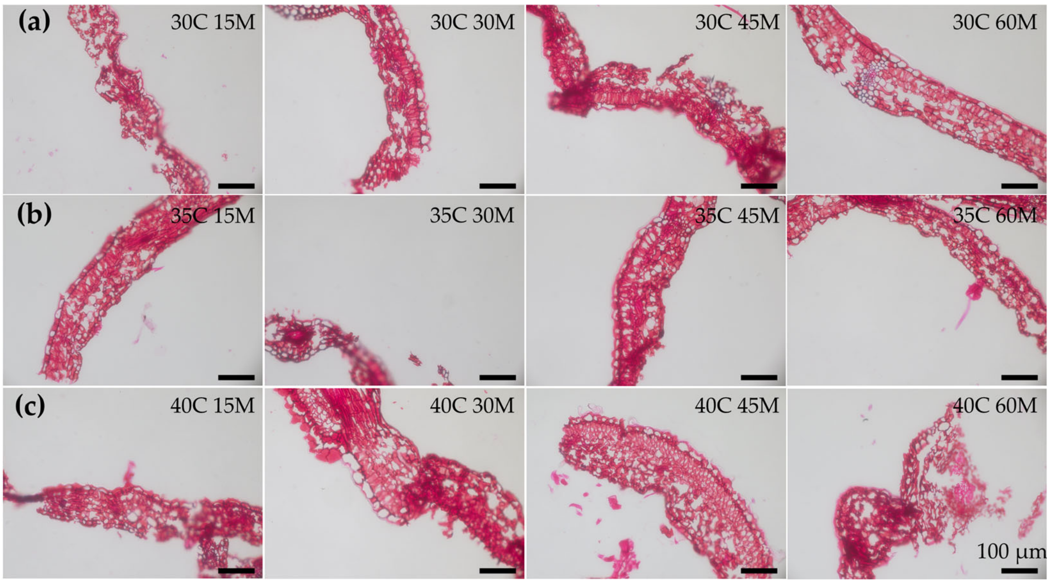


**Figure S2**. Safranin staining of leatherleaf treated at (**a**) 30 °C, (**b**) 35 °C, or (**c**) 40 °C in 5% NaOH for 15, 30, 45, or 60 min, followed by decellularization in 2% SDS and clearing solution ($n$ = 3).
